# Cyclic di-AMP regulates genome stability and drug resistance in *Mycobacterium* through RecA-dependent and RecA-independent recombination

**DOI:** 10.1093/pnasnexus/pgae555

**Published:** 2024-12-12

**Authors:** Sudhanshu Mudgal, Nisha Goyal, Manikandan Kasi, Rahul Saginela, Anusha Singhal, Soumyadeep Nandi, A K M Firoj Mahmud, Kalappa Muniyappa, Krishna Murari Sinha

**Affiliations:** Amity Institute of Biotechnology, Amity University Haryana, Gurgaon, Haryana 122413, India; Amity Institute of Biotechnology, Amity University Haryana, Gurgaon, Haryana 122413, India; Department of Biochemistry, Indian Institute of Science, Bangalore, Karnataka 560012, India; Amity Institute of Biotechnology, Amity University Haryana, Gurgaon, Haryana 122413, India; Amity Institute of Biotechnology, Amity University Haryana, Gurgaon, Haryana 122413, India; Department of Plant Physiology, Umeå Plant Science Centre, Umea University, Umeå 901 87, Sweden; CLINTEC, Karolinska Institutet, Alfred Nobels alle 8, 141 52 Huddinge, Stockholm, Sweden; Department of Biochemistry, Indian Institute of Science, Bangalore, Karnataka 560012, India; Amity Institute of Biotechnology, Amity University Haryana, Gurgaon, Haryana 122413, India

**Keywords:** cyclic di-AMP, cyclic dinucleotides, RecA, DNA recombination, SOS response

## Abstract

In *Escherichia coli*, RecA plays a central role in the rescue of stalled replication forks, double-strand break (DSB) repair, homologous recombination (HR), and induction of the SOS response. While the RecA-dependent pathway is dominant, alternative HR pathways that function independently of RecA do exist, but relatively little is known about the underlying mechanism. Several studies have documented that a variety of proteins act as either positive or negative regulators of RecA to ensure high-fidelity HR and genomic stability. Along these lines, we previously demonstrated that the second messenger cyclic di-AMP (c-di-AMP) binds to mycobacterial RecA proteins, but not to *E. coli* RecA, and inhibits its DNA strand exchange activity in vitro via the disassembly of RecA nucleoprotein filaments. Herein, we demonstrate that *Mycobacterium smegmatis ΔdisA* cells, which lack c-di-AMP, exhibit increased DNA recombination, higher frequency of mutation, and gene duplications during RecA-dependent and RecA-independent DSB repair. We also found that c-di-AMP regulates SOS response by inhibiting RecA-mediated self-cleavage of LexA repressor and its absence enhances drug resistance in *M. smegmatis ΔdisA* cells. Together, our results uncover a role of c-di-AMP in the maintenance of genomic stability through modulation of DSB repair in *M. smegmatis*.

**Significance Statement**

Cyclic di-AMP is a second messenger present in bacteria and archaea and is implicated in a variety of functions in the cell, including DNA repair, cell wall metabolism, virulence, and gene expression. We show here that it maintains genome stability in *Mycobacterium* by regulating RecA-dependent and RecA-independent DNA recombination pathways. It also regulates SOS response by inhibiting the self-cleavage of LexA by mycobacterial RecA. Absence of c-di-AMP leads to higher drug resistance in *Mycobacterium*.

## Introduction

Homologous recombination (HR) is an evolutionarily conserved process that plays a pivotal role in the maintenance of genome integrity in mitotic cells and homologous pairing and DNA strand exchange during meiosis (1, 2). In bacteria, HR is essential for DNA repair, reactivation of stalled replication forks, and acquisition of drug resistance (3–6). Whereas HR in bacteria has been thought to be dependent on RecA-mediated HR pathways, a substantial level of HR occurs independently of RecA in certain genetic backgrounds (7–9). In RecA-mediated HR, RecA protein preferentially binds to single-stranded DNA (ssDNA) to form a right-handed helical nucleoprotein filament, which subsequently promotes homologous pairing and DNA strand exchange (3, 10, 11).

The assembly of RecA nucleoprotein filament is rapid and bidirectional (3, 12), although RecA polymerization is 50–60% faster in the 5′→3′ direction (13), and is tightly regulated by a network of positive (14–20) and negative (21, 22) protein effectors. Of note, mechanical forces regulate the stability of the RecA nucleoprotein filament and can counteract the inhibitory effect of RecX, prevent disassembly of the RecA filament, and stimulate the re-polymerization of RecA on ssDNA despite the presence of RecX (23–25). We previously demonstrated that cyclic di-AMP (c-di-AMP) binds with low micromolar affinity to the C terminus of mycobacterial RecA, but not to *Escherichia coli* RecA, and inhibits its DNA strand exchange via disassembly of RecA nucleoprotein filaments (26).

The bacterial second messenger c-di-AMP, discovered in 2008 (27), is essential for the viability of various bacterial and archaeal species (28–30). It has been implicated in diverse essential cellular processes including central metabolic pathways (31, 32), osmolyte transport (33, 34), cell wall homeostasis and drug resistance (35, 36), progression of sporulation (37), host–pathogen interactions and bacterial virulence (38, 39), and DNA damage repair (26, 40, 41). In mycobacteria, DisA is the sole diadenylate cyclase involved in the production of c-di-AMP and phosphodiesterase DhhP promotes its degradation (42, 43).

Following from our previous observation that c-di-AMP binds to the C terminus of mycobacterial RecA and attenuates its DNA strand exchange activity in vitro through disassembly of RecA nucleoprotein filaments (26), we demonstrate in this study that cells lacking c-di-AMP exhibit: (i) significantly increased levels of homology-directed repair (HDR) and inappropriate HR, (ii) high frequency of deletions and duplications, and (iii) higher level of SOS induction and drug resistance. Our results highlight a previously unappreciated, crucial role for c-di-AMP in the regulation of HR, acquisition of antibiotic resistance, and maintenance of genome stability in *Mycobacterium smegmatis*.

## Results

### Absence of c-di-AMP potentiates HDR of DSB in *M. smegmatis*

**Fig. 1.**
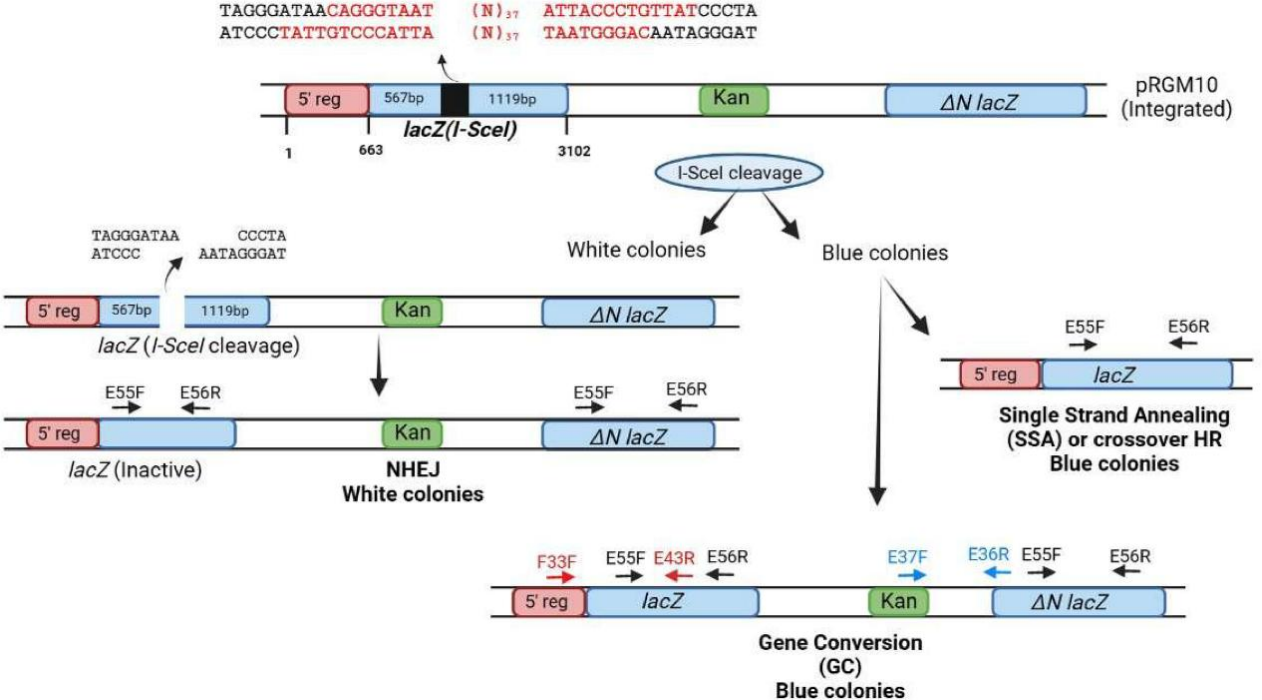
Reporter construct pRGM10 and the known repair pathways and outcome: the upper panel shows the reporter construct pRGM10 integrated at the *attB* site of *M. smegmatis* cells. The sequences written above pRGM10 are the two 18-mer I-SceI target sequences placed 37 nt apart in the opposite orientation in *lacZ*(*I-SceI*). The sequences shown in black are of the DSB generated with 3′-overhangs after cleavage by I-SceI. The break will be repaired by either NHEJ or HR giving rise to scorable white and blue colonies, respectively. The repair outcomes were analyzed by performing PCR amplification with diagnostic primers. The position and orientation of the primers with their numbers have been shown with arrows to indicate their orientation on the repaired (outcome) sequence. PCR with primers E55F and E56R will give a product of 1,311 bp on repair by GC or SSA (blue colonies), whereas repair by NHEJ will give two PCR products of 1,311 and 779 bp or smaller as per the number of nucleotides deleted. E37F and E36R were used to detect kanamycin gene (Kan) to distinguish between GC and SSA. Primers F33F and E43R were used to analyze the repair outcome by detecting the presence of the 5′ *lacZ* DNA sequence.

**Fig. 2.**
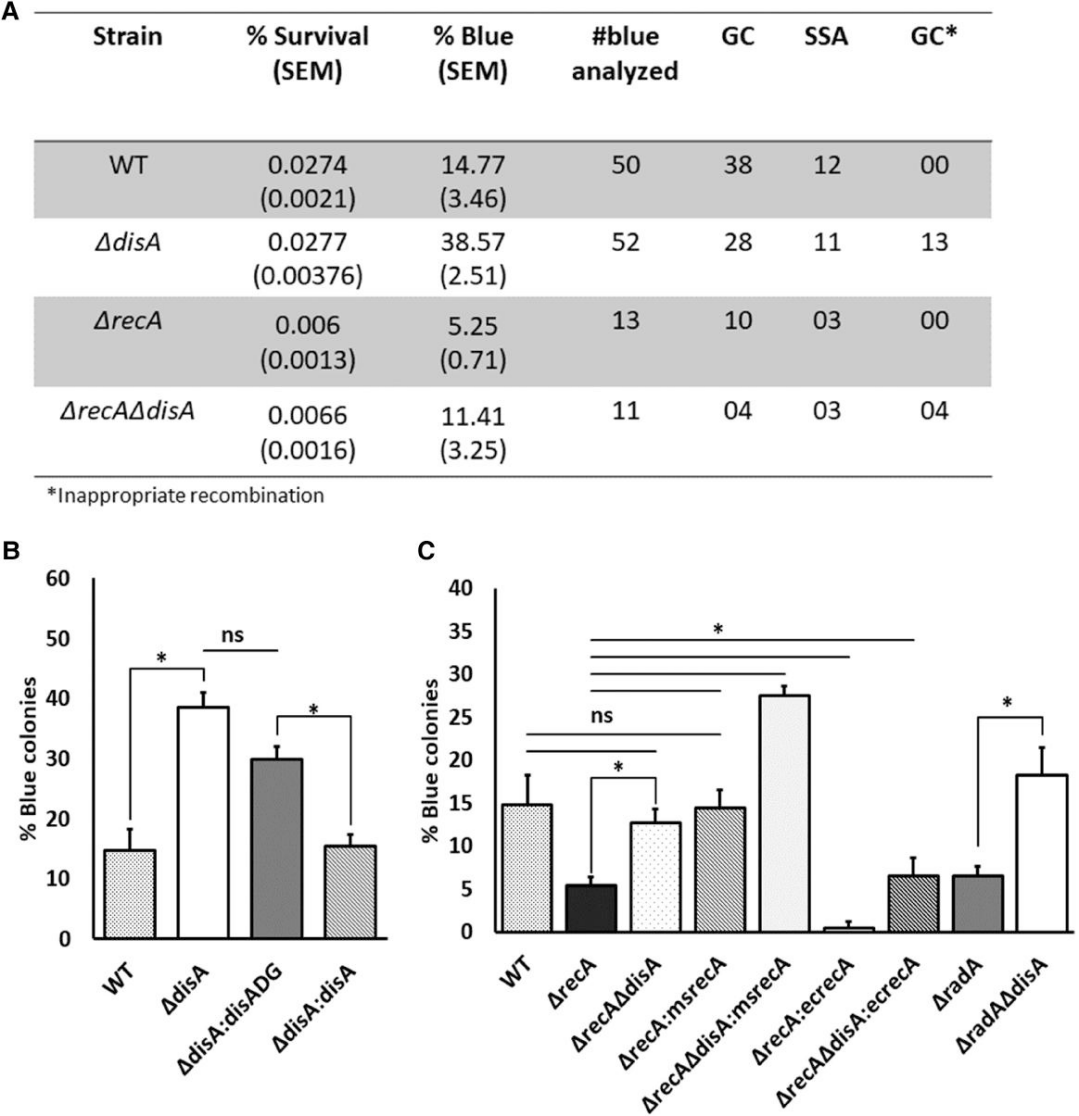
Absence of c-di-AMP leads to higher HR: A) The table shows the % survival and % blue colonies (with SEM) of the different mycobacterial strains after repair of I-SceI-induced DSB. The number of blue colonies analyzed to determine the repair outcome (#blue analyzed) and the repair by GC, SSA, and GC* as determined has been given. GC* represents the number of blue colonies obtained after the repair of DSB by NHEJ as explained in the results. B) Bar graph showing the percentage of blue colonies obtained in WT, *ΔdisA*, *ΔdisA:disADG* (*ΔdisA* complemented with active site mutant DisA) and *ΔdisA:disA* (*ΔdisA* complemented with wild-type DisA). The percentage of blue colonies was calculated as: number of blue colonies/total survivors on the plate × 100. The difference among the blue colonies in different strains was found to be statistically significant (*P ≤ 0.05). C) Percentage of blue colonies obtained in WT, *ΔrecA*, *ΔrecAΔdisA*, *ΔrecA:msrecA* (*ΔrecA* complemented with MsRecA), *ΔrecAΔdisA:msrecA* (*ΔrecAΔdisA* complemented with MsRecA), *ΔrecA:ecrecA* (*ΔrecA* complemented with EcRecA), *ΔrecAΔdisA:ecrecA* (*ΔrecAΔdisA* complemented with EcRecA), *ΔradA*, and *ΔradAΔdisA*. Each experiment was performed with at least three biological replicates, and each replicate was performed in triplicate.

*Mycobacterium* employs three pathways for double-strand break (DSB) repair: nonhomologous end joining (NHEJ), HR, and single-strand annealing (SSA). To gain insight into the relative contributions of these pathways to DSB repair in mycobacteria, the Shuman and Glickman group had developed a reporter system in which I-SceI-induced DSB repair could be studied genetically and mapped physically (44). To elucidate the physiological relevance of c-di-AMP during DSB repair in *M. smegmatis*, we leveraged the same I-SceI-based reporter assay (Fig. 1). In this genetic assay, repair of DSB yields blue and white colonies: white colonies can result from NHEJ-mediated repair or I-SceI inactivating mutation and LacZ^+^ (blue) colonies from error-free HR or SSA repair pathway (Fig. 1). Our results revealed that the frequency of LacZ^+^ colonies in the mutant *ΔdisA* strain was 2.6 times higher as compared to the WT strain: this implies that increased HR-mediated DSB repair is likely due to the absence of c-di-AMP (Fig. 2A). These results are also consistent with the results of our previous study where we had shown that c-di-AMP attenuates the DNA strand exchange activity of mycobacterial RecA (26). *ΔdisA M. smegmatis* cells had undetectable level of RecA initially, but the expression came back to the WT level with time and has been used in the present study (Fig. S1C–E).

To determine whether the re-expression of RecA is because of some mutation in the promoter or neighboring region, we sequenced 1.0 kb upstream and downstream of *recA* gene (*msmeg_2723*) but did not find any mutation. The mutation has likely occurred in the genome affecting a transcription factor or a component of ribosome, rRNA, ribosomal proteins (45), or sRNA (46) causing the change in the expression of RecA. The possibility of a reverse mutation cannot be ruled out either. A conclusive answer about the re-expression can be obtained after analyzing WGS data. Determination of SNPs in the genome sequence of *ΔdisA* cells identified mutations in several genes including transcriptional regulators, tRNA-modifying enzymes, DNA endonuclease, etc. (Table S1). Suppressor mutations are common in *ΔdisA* cells, and this has led to the discovery of several pathways regulated by the dinucleotide (47, 48). We also determined the sensitivity of the *ΔdisA M*. *smegmatis* cells to different DNA clastogens, viz. UV, MMS, H_2_O_2_, rifampicin, and ofloxacin, to assess the changes in *ΔdisA* cells because of the RecA re-expression (Fig. S2). We consistently found the *ΔdisA* cells to be more sensitive to the DNA clastogens, suggesting a role of c-di-AMP in DNA damage repair.

To test whether the increased HR-mediated DSB repair was due to the lack of c-di-AMP and not DisA protein, *ΔdisA* cells were complemented with either WT *disA* or catalytically inactive *disA*^D72A/G73A^ variant (designated as DisADG) (42) and the repair efficiency was determined by counting LacZ^+^ colonies. We observed that the percentage of LacZ^+^ colonies in the *ΔdisA* and *disADG* strains was similar; it increased 2-fold as compared to the WT (Fig. 2B). On the contrary, the percentage of LacZ^+^ colonies in *ΔdisA* cells bearing WT *disA* allele was similar to that of the wild-type cells. Collectively, these results suggest that c-di-AMP not DisA negatively affects HR-mediated DSB repair *in M. smegmatis.*

### c-di-AMP regulates both the RecA-dependent and RecA-independent HR

Given that the deletion of *disA* led to an increase in the amount of LacZ^+^ colonies, we asked whether the increase in LacZ^+^ colonies is because of higher DNA strand exchange activity of RecA in the absence of c-di-AMP as predicted from our previous work (26). For this purpose, *M. smegmatis ΔrecA* and *ΔrecAΔdisA* knockout mutants were constructed and their ability to form LacZ^+^ colonies was compared with that of *ΔdisA* cells. We reasoned that if the increase in LacZ^+^ colonies is because of RecA, *ΔrecA* and *ΔrecAΔdisA* cells will have a reduced number of such colonies. Consistent with our premise, a 3.4-fold decrease in the number of blue LacZ^+^ colonies was observed in the double mutant *ΔrecAΔdisA* strain than that in *ΔdisA* cells, suggesting that RecA-mediated HR pathway is involved in the formation of blue LacZ^+^ colonies in *ΔdisA* cells (Fig. 2B and C). It is interesting to note that blue colonies were obtained even in the absence of RecA in *ΔrecAΔdisA* cells, suggesting the presence of RecA-independent HDR (8, 9, 49). This result was also confirmed in *ΔrecA* cells where blue colonies were obtained though the percentage decreased significantly (2.8-fold) compared with the WT *M. smegmatis* cells (Fig. 2A and C). Interestingly, the percentage of blue colonies in *ΔrecAΔdisA* double mutant is 2.2-fold higher than in *ΔrecA* cells (Fig. 2C), suggesting that c-di-AMP also regulates RecA-independent homology-mediated DSB repair.

Complementation of *ΔrecA* and *ΔrecAΔdisA* cells with cognate *M. smegmatis* RecA (MsRecA) increased the HDR to WT and *ΔdisA* levels, respectively, but complementation with *E. coli* RecA (EcRecA) did not increase the percentage of HDR. The extent of homology-mediated repair in EcRecA complemented cells was as less as the parent cells (Fig. 2C), suggesting that EcRecA is not functional in *M. smegmatis*.

As *disA* and *radA* exist in an operon and this locus is conserved across several bacterial species, we studied the role of RadA in HR. Moreover, RadA induces heteroduplex branch migration activity of RecA in *E. coli* (50), prompting us to speculate that RadA is involved in c-di-AMP-mediated RecA-dependent HR in *Mycobacterium*. To find out the role of RadA and the regulation of its activity by c-di-AMP, we created *ΔradA* and *ΔradAΔdisA M. smegmatis* cells and determined the repair of DSB as above. The percentage of HR as determined by the number of LacZ^+^ colonies obtained decreased to 6.6% in *ΔradA* cells as compared to 15% in the WT cells, whereas the percentage increased to 18% in *ΔradAΔdisA* cells, corroborating the above result of the role of c-di-AMP in HR. Further work is required to reveal the function of RadA in detail and its regulation by c-di-AMP.

### Deletion of *disA* does not affect NHEJ repair

Next, white colonies were sequenced to decipher the role of c-di-AMP on DSB repair promoted by NHEJ. The DSB repair junctions were amplified with forward and reverse primers, E55F and E56R (Fig. 1), and PCR products were sequenced to detect the NHEJ-mediated repair outcome. Of the 30 representative white-colored colonies sequenced for the WT and *ΔdisA* strain each, 52% were repaired through NHEJ and 48% had intact I-SceI site suggesting inactivation of the *I-SceI* gene through mutation (44). Both the I-SceI target sequences were cleaved in all the NHEJ colonies, and most of the repair was done by deleting nucleotides at the break site to expose homology at the ends followed by ligation. The deletions were mostly of a few nucleotides, and they were similar in both the WT and *ΔdisA* cells (Fig. S3A and B). The surprising outcome was the absence of NHEJ in *ΔrecAΔdisA* cells. We sequenced 20 colonies: 19 were found to have I-SceI inactivation and 1 colony had an insertion of five nucleotides at the 3′ end (Fig. S3C). So, there is a possibility that c-di-AMP/DisA might have indirect effect on NHEJ or impact NHEJ-mediated repair under certain conditions.

### Deletion of *disA* results in hyper-recombinogenic RecA protein

**Fig. 3:**
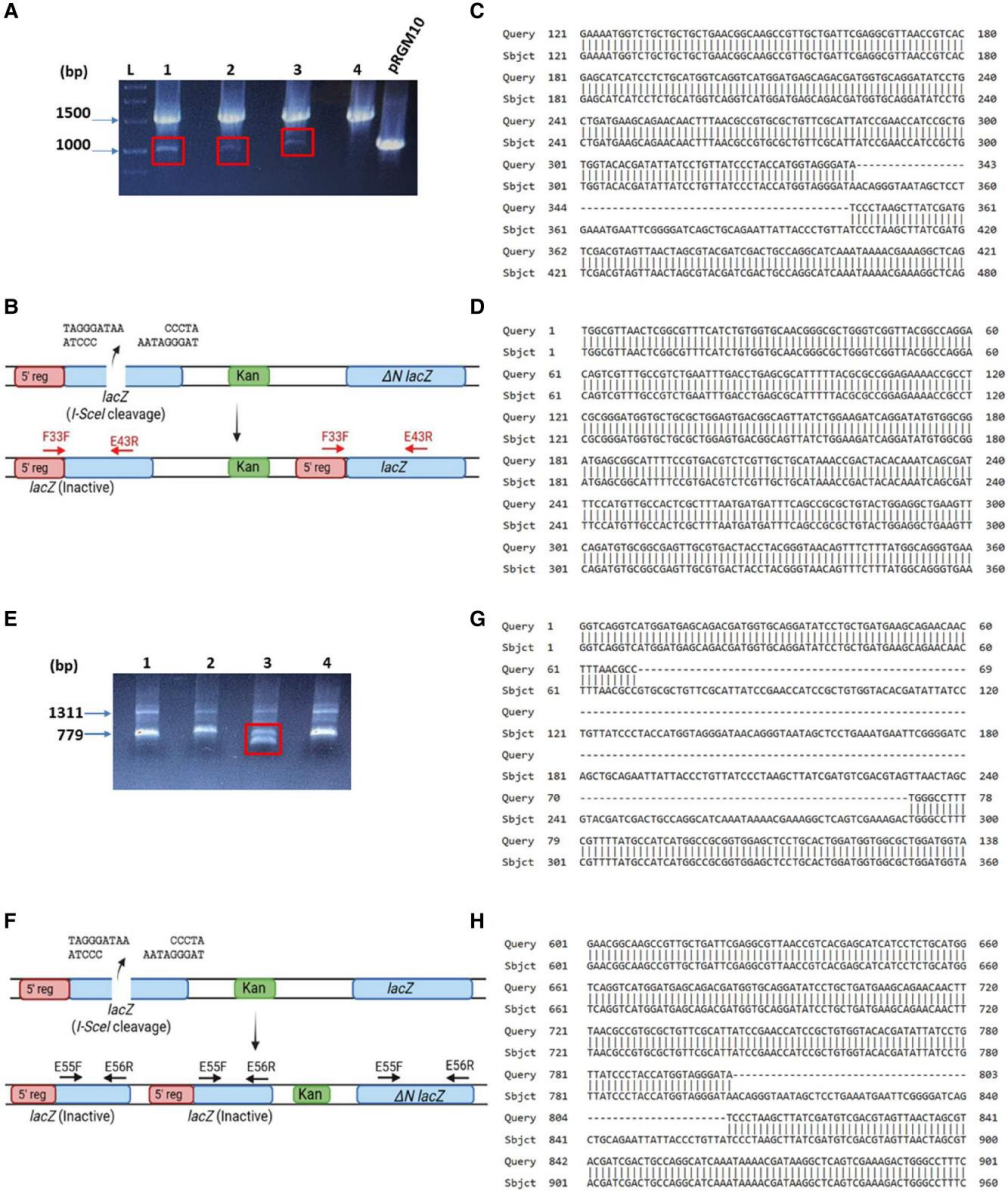
. Hyper-recombination and gene duplication in *ΔdisA* cells: A) Repair events of four representative blue colonies (numbered 1–4) have been shown. #1–3 have undergone repair by GC*, whereas #4 is repaired by GC after I-SceI cleavage. The colonies were analyzed by PCR amplification with primers F33F and E43R shown in B). PCR products were separated on an agarose gel and ethidium bromide-stained gel has been shown. A major product of 1,507 bp was obtained along with an additional smaller product (shown in red box). pRGM10 was used as a control template. Arrows on the left side of the gel show the size and position of the bands. B) The repair outcome of the blue colonies #1–3. The break has been repaired by NHEJ, but GC has also occurred transferring the 5′ 662nt to *ΔNlacZ* giving rise to active *lacZ*. C) The smaller product results from nucleotide deletion across the break site during NHEJ. The figure shows alignment of the sequence of smaller PCR product of colony #1 with that of the *lacZ-I-SceI* sequence. The gaps in the alignment arise because of nucleotide deletion during NHEJ repair of DSB. D) Sequence of the bigger PCR product (with primers F33F and E43R) confirms the recombination of 5′ 662nt to *ΔNlacZ*. E) Repair events in four representative white colonies after I-SceI expression were analyzed by PCR amplification using primers E55F and E56R. Two products of 1,311 and around 779 bp were obtained as expected (as explained in Fig. 1), but there was an additional smaller product in colony #3. Both the smaller bands of colony #3 have been marked with red box. Arrows on the left indicate the size and position of the bands. F) The repair outcome in colony #3. There is duplication of the lacZ(I-SceI) gene after repair by NHEJ. G) and H) The sequence alignment of the smaller PCR products with the lacZ gene has been shown. The gaps indicate deletion of different lengths.

The function of RecA is tightly regulated by a complex network of positive and negative modulators that act to maintain a balance between inappropriate and appropriate recombination events (3, 11, 21, 51). As c-di-AMP attenuates mycobacterial RecA-promoted DNA pairing and strand exchange reactions (26), we reasoned that RecA may display a characteristic “hyper-rec” phenotype with aberrant recombination events in *ΔdisA* cells. To test this hypothesis, the repair outcome across I-SceI junction in *lacZ(I-SceI)* gene in blue colonies was PCR-amplified with the primers F33F and E43R (Fig. 3B). In all the cases, a major PCR product migrating at the expected size of 1,507 bp was observed; this results from the DSB repair via gene conversion (GC) or SSA (44). Unexpectedly, however, an additional band of about 780 bp was seen in 25% of blue LacZ^+^ colonies (Fig. 3A). Sequencing showed that the 780-bp PCR product originated from NHEJ-repaired *lacZ(I-SceI)* gene (Fig. 3C). As the DSB repair by NHEJ should produce white colonies, the 1,507-bp PCR product was also sequenced to decipher the possible mechanism behind the formation of blue LacZ^+^ colonies (Fig. 3D). The results revealed that the 662-bp region located near the 5′ end of *lacZ(I-SceI)* gene has recombined with the downstream (*ΔNlacZ*) gene giving rise to blue LacZ^+^ colonies in this assay. Although this recombination event is similar to the repair of DSBs by GC, we posit that the DSB was first repaired by NHEJ, followed by HR by “hyper-rec” RecA, which differs from the previous observations (44).

We also interrogated whether such NHEJ-repaired blue colonies were obtained in *ΔrecA* and *ΔrecAΔdisA* strains. To this end, 13 and 11 blue colonies from strains with a single *ΔrecA* mutation and *ΔrecAΔdisA* double mutations, respectively, were sequenced. Of these, 36% of blue LacZ^+^ colonies in *ΔrecAΔdisA* strain were repaired by NHEJ followed by HR between the 5′ end of *lacZ(I-SceI)* and *ΔNlacZ*, but no such recombination events occurred in the *ΔrecA* or WT strains. This result along with the results in the previous section clearly suggests that the absence of c-di-AMP leads to LacZ^+^ blue colonies through inappropriate recombination in *ΔdisA* and *ΔrecAΔdisA* cells. The inappropriate recombination is because of “hyper-rec” RecA in Δ*disA* cells, but it is RecA-independent in *ΔrecAΔdisA* cells. These results also show that a significant percentage of HR events can proceed independently of RecA in *M. smegmatis* and that c-di-AMP inhibits both the RecA-dependent and RecA-independent HR, although the precise mechanism remains unclear.

The outcome of DSB repair in white colonies was also assessed by PCR amplification of their genomic DNA using primers E55F and E56R (Fig. 1A). As expected, two PCR products migrating at the expected size of 1,311 and 779 bp or smaller depending on the deletion during NHEJ were observed. In one of the 30 white colonies analyzed from the *ΔdisA* strain, we observed the 1,311-bp product but got two smaller products instead of one (Fig. 3E). Sequencing of the smaller products suggested that they were generated because of NHEJ deletion (Fig. 3G and H). We reasoned that the second product would appear either because of duplication of the first repaired gene or a mixed colony. To rule out the second possibility, we streaked the culture of the colony and determined the repair outcome in six individual colonies by performing genomic PCR as above (Fig. S4A). All six colonies gave two smaller products as above, suggesting that the colony was not mixed but there was a duplication of *lacZ(I-SceI)* gene.

### Deletion of *disA* enhances the SOS response and promotes drug resistance

**Fig. 4:**
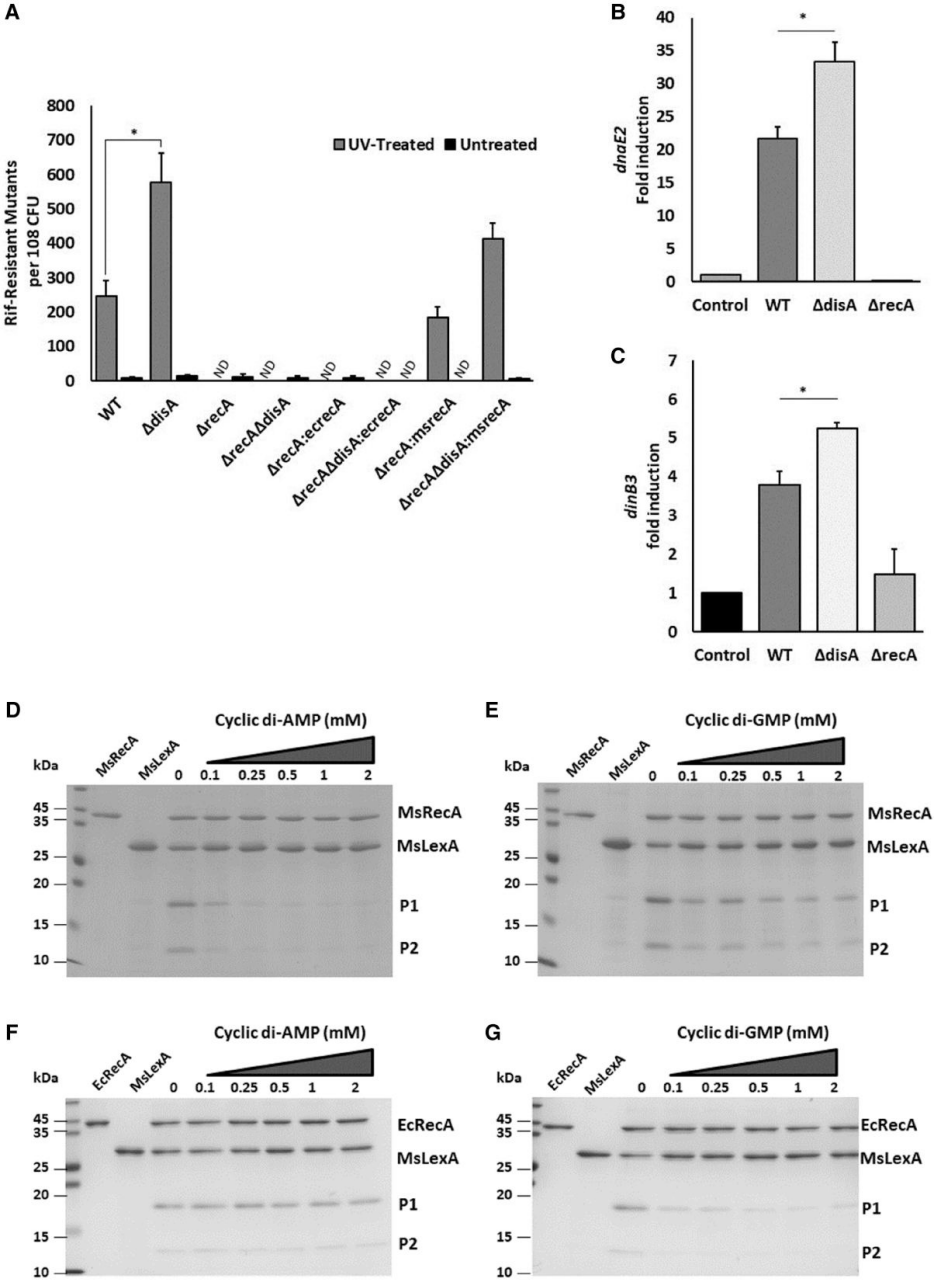
. Absence of c-di-AMP induces stronger SOS response. A) Frequency of rifampicin-resistant mutants per 10^8^ cells in untreated (black bars) or UV-treated (gray bars) of the indicated *M. smegmatis* strains. ND, not detected. B and C) Transcript levels of *dnaE2* and *dinB3* in WT, *ΔdisA,* and *ΔrecA M. smegmatis* cells after treatment with quinolones to induce SOS response. *P < 0.05 for indicated comparisons by paired t test. Error bars represent SD. All experiments were performed with three biological replicates, and each replicate was performed in triplicate. D–G) Inhibition of LexA cleavage by c-di-AMP and c-di-GMP; D) and E) show the cleavage of MsLexA by MsRecA in the presence of c-di-AMP and c-di-GMP, respectively. MsLexA was incubated with MsRecA in the presence of increasing concentration of c-di-AMP (C) and c-di-GMP (D) as described in the methods. The reaction mixture was run on SDS–PAGE, and the Coomassie-stained gel has been shown. The first two lanes show the purified MsRecA and MsLexA recombinant proteins as labeled at the top. The different concentration of the dinucleotides used in the reaction varies from 0 to 2 mM as mentioned at the top of the lanes. The position and size of protein ladder have been marked at the left. P1 and P2 (marked on the right) are the two cleavage products of MsLexA. F) and G) show the cleavage MsLexA with EcRecA in the presence of c-di-AMP and c-di-GMP, respectively, as shown at the top of the figures. The cleavage assay was carried out as above, and Coomassie-stained SDS–PAGE gel has been shown.

Most bacteria utilize the tightly regulated RecA/LexA SOS response system to induce DNA repair genes, wherein activated RecA stimulates the autocleavage of LexA repressor (52). Mycobacteria harbor at least two DNA damage response pathways: the LexA/RecA-dependent SOS response (53–56) and the PafBC-regulated pathway (57–59). We have demonstrated previously that c-di-AMP binds to the C terminus of mycobacterial RecA and inhibits DNA strand exchange through disassembly of RecA nucleoprotein filaments (26). Hence, we postulated that *ΔdisA* cells possessing hyper-recombinogenic RecA may cause stronger SOS induction resulting in mutation and drug resistance. To this end, the SOS response was assessed in the *M. smegmatis ΔdisA* cells after UV irradiation and growth on rifampicin (60). We found a 2-fold increase in the number of rifampicin-resistant colonies in the *ΔdisA* mutant as compared to the WT, whereas rifampicin-resistant colonies were undetectable in *ΔrecA* and *ΔrecAΔdisA* knockout mutants (Fig. 4A): this implies that the hyper-rec activity of RecA induces a hypermutable state that promotes acquisition of resistance to rifampicin (58, 60). Furthermore, complementation of *M. smegmatis ΔrecA* mutant with its cognate WT *recA*, but not *E. coli recA*, conferred rifampicin resistance. Similarly, *ΔrecAΔdisA* knockout mutant complemented with its cognate WT *recA* formed a higher number of rifampicin-resistant colonies, indicating a role for c-di-AMP in the SOS response and mutagenesis (Fig. 4A). In contrast, *ΔrecA* and *ΔrecAΔdisA* mutants complemented with *E. coli recA* did not induce the SOS response (Fig. 4A), as *E. coli* RecA is inactive in *Mycobacterium*.

In mycobacteria, trans-lesion synthesis DNA polymerases, DnaE2 and DinB, enable cells to confer rifampicin resistance (60, 61). During SOS response, *dnaE2* is induced in a RecA-dependent manner, although the intrinsic/extrinsic factors involved in the induction of *dinB* remain unclear. To gain insights into the mechanism underlying acquisition of drug resistance by *ΔdisA* mutant, we monitored the induction of *dnaE2*, *dinB1, dinB2,* and *dinB3* in response to 10 µg/mL of ofloxacin treatment, followed by qPCR. The results show a 20- and 35-fold increase in the transcript levels of *dnaE2* in WT and *ΔdisA* mutant, respectively (Fig. 4B). On the contrary, a 3.7- and 5.3-fold increase in the *dinB3* transcript levels was observed in the WT and *ΔdisA* cells, respectively, whereas no significant change was observed in *dinB1* and *dinB2* transcript level.

Next, we sought to characterize the biochemical basis of the SOS-induced mutagenesis in the *ΔdisA* mutant. To this end, MsRecA-mediated autocleavage of its cognate LexA repressor was monitored in the absence and presence of c-di-AMP. The reaction products were analyzed by SDS–PAGE and visualized by staining with Coomassie blue. It revealed that c-di-AMP, but not c-di-GMP, abrogates MsRecA-mediated autocleavage of LexA repressor in a concentration-dependent manner (Fig. 4C and D). In contrast, c-di-GMP inhibited EcRecA-mediated autocleavage of *M. smegmatis* LexA repressor, but c-di-AMP did not (Fig. 4E and F).

## Discussion and perspectives

DNA integrity scanning protein or DisA was discovered as a protein that scans the chromosome during sporulation in *B. subtilis* and blocks sporulation on detecting any damage (62). The DAC activity of DisA is inhibited on binding to Holliday junction or replication fork-like structures (27). *B. subtilis* and *Mycobacterium* contain a conserved operon consisting of *disA* and *radA* genes, suggesting a role of DisA/c-di-AMP in DNA damage repair. Our previous work has demonstrated that c-di-AMP binds to the C terminus of mycobacterial RecA, but not EcRecA, and attenuates DNA strand exchange activity of MsRecA in vitro (26). Here, we show that *M. smegmatis ΔdisA* strain, which lacks c-di-AMP, exhibits increased HDR levels of RecA-dependent and RecA-independent DSB repair, although the precise role of c-di-AMP in latter pathway remains unclear, warranting further investigation. Of note, *ΔdisA* null mutant displays hyper-recombination and hypermutable phenotype, which are important mechanisms responsible for acquired drug resistance in bacteria. Further analysis of *ΔdisA* mutant revealed that it exhibits a higher frequency of deletions and duplications and a 2-fold increase in the acquisition of rifampicin resistance. Moreover, we found that the loss of c-di-AMP results in robust SOS response via inhibition of RecA-mediated self-cleavage of LexA repressor. The inhibition of LexA cleavage in both *Mycobacterium* and *E. coli* suggests that the regulation of SOS response by cyclic dinucleotides could be a general phenomenon. Together, our results uncover a role of c-di-AMP in the maintenance of genomic stability through modulation of DSB repair in *M. smegmatis*.

**Fig. 5:**
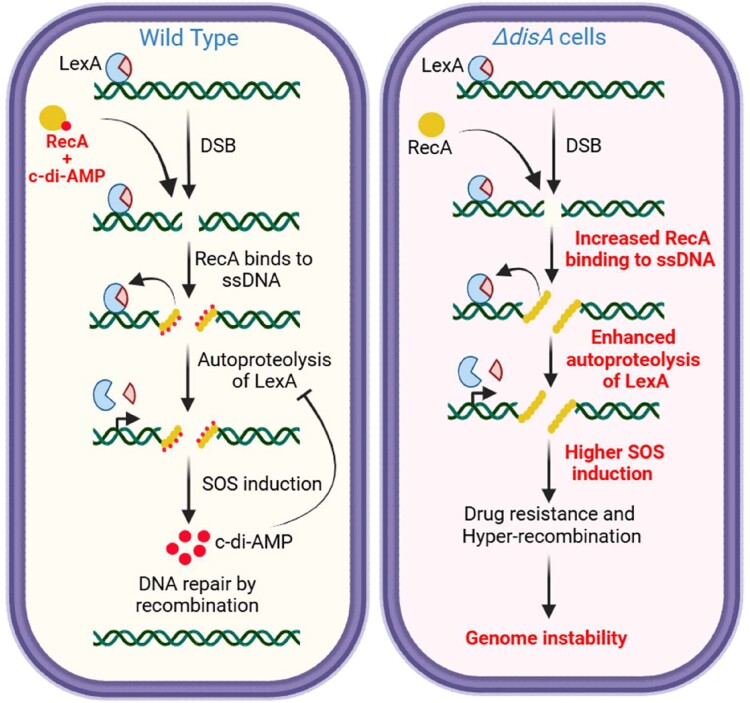
. Model showing the function of c-di-AMP in RecA-dependent HDR and SOS response. c-di-AMP binds to mycobacterial RecA and regulates its DNA strand exchange activity and nucleoprotein filament formation. In the absence of c-di-AMP, there are higher DNA recombination and increased SOS induction. Higher SOS induction leads to enhanced genome mutation and drug resistance. Higher DNA recombination causes illegitimate recombination, gene duplication, etc., leading to genome instability.

Although considerable progress has been made toward understanding the role of accessory proteins that negatively regulate RecA-mediated HR (11, 19, 21), our knowledge about the potential roles of endogenous small molecule inhibitors, including bacterial second messenger(s), in the regulation of RecA function is limited. In this context, it is worth highlighting that c-di-AMP, but not c-di-GMP, inhibits the recombination-like activities of mycobacterial RecA proteins, but not the EcRecA, in vitro (26), consistent with the in vivo data reported in this study. The combined in vitro and in vivo data provide valuable insights into the molecular basis by which c-di-AMP regulates RecA activities and DNA repair/recombination functions. Taking into consideration the effects of c-di-AMP in vivo and in vitro, we propose a model as to how it may regulate genomic stability in *M. smegmatis* (Fig. 5). The absence of c-di-AMP promotes the formation of RecA nucleoprotein filament and causes RecA to become hyperactive. The hyperactive RecA results in higher and illegitimate recombination and enhanced SOS induction. Higher SOS response induces the expression of Y-family of DNA polymerases, DnaE2 and DinB3, leading to genome mutation (including the suppressor mutations) and drug resistance. It is interesting to note that *disA* transcript is induced as a result of SOS response and c-di-AMP so synthesized inhibits SOS induction most probably through a feedback mechanism. Our postulated concept outlined in this model may help to stimulate further investigations into how c-di-AMP can differentially affect RecA-dependent and RecA-independent DSB repair pathways to decipher whether it plays a direct, indirect, or additive role in vivo. Given the presence of cyclic dinucleotides in all domains of life, their role in maintaining genome stability might be conserved. There are suggestions for the involvement of cGAS in genome stability in metazoans (63).

Bacterial resistance to antibiotics is a major global health concern, which poses a serious and rapidly growing threat to human, animal, and environment health (64). The enzymes involved in DNA repair/recombination have been linked to the emergence of drug-resistant pathogenic bacteria, especially multidrug-resistant and extensively drug-resistant TB strains (65). Consequently, attenuation of the SOS response system via preferential inhibition of RecA has been proposed as a possible therapeutic target to block the development of multidrug resistance and resistance-conferring mutagenesis (66, 67). Several inhibitors of RecA activities in vitro have been developed including ATP-competitive nucleotide analogs (68, 69), polysulfated naphthyl compounds (70–72), as well as short peptides (73). To our knowledge, none of these synthetic small molecules have demonstrated RecA-specific biological activities in bacterial cell cultures. This work provides a framework for designing or re-engineering endogenous small molecules with enhanced activity against bacterial RecA to reduce the acquisition of antibiotic resistance mutations and evolution of drug-resistant bacteria.

## Materials and methods

Reporter construct pRGM10- and I-SceI-mediated DSB: Reporter construct pRGM10 was used to study I-SceI-mediated DSB repair in this study (44). pRGM10 contains two functionally inactive *lacZ* genes which are placed 2.1 kb apart with a kanamycin gene in between (Fig. 1). The upstream *lacZ(I-SceI)* gene has an internal deletion of 745 bp in the *lacZ* open reading frame (ORF) and is replaced with two oppositely oriented I-SceI target sequences inserted 37 bp apart. The downstream (*lacZ-ΔN*) gene does not have 5′ 662 nt coding sequence of the *lacZ* ORF, but it has the deleted region of *lacZ(I-SceI)*. The two *lacZ* genes have homologous regions of 567 and 1,119 bp upstream and downstream of the DSB break, respectively. The construct was integrated at the *attB* site of *M. smegmatis* chromosome by electroporating pRGM10 along with pBSIntegrase which contains the L5 integrase gene (26). Cells were plated in the presence of 20 µg/mL of kanamycin on 7H10 agar plates supplemented with 0.5% glycerol and 10% OADC. Integration was confirmed by performing PCR with suitable primers. The forward primer (D69F) anneals 5′ to the *attB* site in the chromosome, whereas the reverse primer E35R anneals 5′ to *lacZ(I-SceI)* gene within the construct.

ORF of *I-SceI* was amplified from pMSG375 using the primers E16F and E17R and cloned between SphI-EcoRI site of pVV17 to give the plasmid pVVI-SceI. To induce DSB, *M. smegmatis* cells containing pRGM10 were electroporated with pVVI-SceI and the colonies were selected against (100 µg/mL) hygromycin on 7H10 agar plates containing 40 µg/mL of X-gal. Expression of I-SceI from a plasmid cleaves at the two target sequences placed in the *lacZ(I-SceI)* gene giving a DSB with noncompatible 3′ overhangs at the two ends. The break can be repaired either by NHEJ or through a homology-mediated repair using (*lacZ-ΔN*) as a template. Repair by NHEJ involves insertion and deletion of nucleotides at the break site and will give rise to kanamycin resistant white colonies, whereas HR by GC or SSA will lead to blue colonies on X-gal plate (44). White colonies are also obtained because of inactivating stochastic mutation in the *I-SceI* gene. The blue colonies obtained after repair by GC are kanamycin resistant, whereas the ones repaired by SSA are kanamycin sensitive (Fig. 1). The number of white and blue colonies obtained is indicative of the extent of repair by NHEJ and HR (or HDR), respectively. Outcome of the repair was determined by performing genomic PCR with suitable primers across the junction and sequencing the PCR product. The genomic DNA of blue colonies obtained because of the repair by GC or SSA was PCR-amplified with forward primer that anneals within the first 662 nt of *lacZ* ORF (F33F) and the reverse primer E43R anneals downstream of the break site (Fig. 1). This set of primers will give a single PCR product of 1,507 bp. The repair outcome of GC and SSA was distinguished by performing PCR with kanamycin-specific primers E37F and E36R which will give a PCR product of 1,442 bp for repair by GC.

The repair outcomes of white colonies were determined by sequencing the PCR product amplified by diagnostic primers that anneal across the junction. The colonies with intact I-SceI site were sequenced to confirm mutations in *I-SceI* gene by amplifying the gene with suitable primers.

### Complementation of the mutant cells

The complementation constructs in this study have been made in the plasmid pYS2 and integrated at the intergenic region of *msmeg_5848* and *msmeg_5849* (44) through DNA recombineering as described before (74). The construct contains 5′ and 3′ homologous sequences for integration, and a cassette containing the gene to be expressed is placed in between the homologous sequences (Fig. S3). The 5′ homologous region containing the last 721 nt of *msmeg_5848* was amplified from *M. smegmatis* genome using the primers F23F and F24R, digested with SpeI and SwaI and cloned in similarly digested pYS2. 3′ homologous region containing the terminal 726 bp of *msmeg_5949* was similarly PCR-amplified with primers F58F and F59R, digested with NsiI and PacI and cloned in similarly digested plasmid construct containing the 5′ homologous region.

The genes to be expressed were first placed under hsp promoter by cloning them initially in pVVgfp(5) (26). The gene cassettes were then amplified from these constructs with the forward primer F19F that anneals at the beginning of the hsp promoter and a gene-specific reverse primer that anneals over the termination of the ORF. The reverse primers used to amplify *msrecA*, *ecrecA*, and *ecmsrecA* are F22R, F21R, and F22R, respectively. Both the forward and the reverse primers contain SwaI site. The PCR product thus obtained contains the hsp promoter along with the gene ORF, digested with SwaI and cloned at the SwaI site of the construct containing the 5′ and 3′ homologous regions. The construct places the gene 5′ to the *gfp-hyg* cassette which is flanked by loxP on either side. The plasmid was digested with SpeI and NsiI to release the complementing construct. The construct was electroporated in *M. smegmatis* cells (either WT or different mutants) which expresses Che9c 60–61 from the plasmid pYS1. The electroporated cells were plated on 7H10 containing hygromycin and sucrose and grown at 42 °C to cure them of pYS1. The cells so obtained have the complementing construct integrated at the intergenic region of *msmeg_5848* and *msmeg_5849* and were screened based on the expression of GFP. The integration and orientation of the gene were further confirmed by performing PCR with primers F48F and F49R (Fig. S3B). The complemented cells were then transformed with pML2714 which expresses P1 Cre recombinase to remove *gfp-hyg* cassette. pML2714 was subsequently removed by growing the cells at 42 °C. The expression of the genes was confirmed by western blotting (Fig. S3C–E).

### UV-induced mutagenesis

For UV-induced mutagenesis, cells were grown in 7H9 medium until saturation. The cultures were then diluted to OD_600_ = 0.02 and grown further to OD_600_ = 0.5. 5.0 mL of the cultures was transferred to 90 × 15 mm sterile plastic petri plates and exposed to 20 mJ/cm^2^ UV radiation using a UVP CL 1000 Ultraviolet Crosslinker. The treated cells were transferred to 5 mL of fresh 7H9 media and grown in a shaker incubator at 37 °C with shaking at 150 rpm for 3 h. The untreated control of each strain was processed similarly. 100 µL of cells from each sample was plated in duplicate on 7H10 agar plates containing 10% OADC, 0.5% glycerol, and 80 µg/mL of rifampicin, and plates were wrapped in a foil to prevent potential effects of photolyase and incubated at 37 °C for 72 h to determine rifampicin-resistant CFU. Cells were taken from each sample, and appropriate dilutions were plated on 7H10 agar plates containing 10% OADC and 0.5% glycerol in the absence of antibiotic to determine viable CFU. The resistant mutants obtained on rifampicin plates were normalized to viable CFU. Each set of experiments was performed with three biological replicates; each replicate was performed in triplicate.

Change in the transcript level of DnaE2 and DinB polymerases during SOS induction was assessed by performing RT-qPCR. *M. smegmatis* cells were grown to OD_600_ ≈ 0.5 and treated with 10 µg/mL of ofloxacin for 3 h. The cells were harvested by centrifugation, and the cell pellet was re-suspended in RNAprotect bacteria reagent (Qiagen) and incubated at RT for 5 min. The cells were re-centrifuged, and the pellet was used for total RNA extraction using RNeasy kit (Qiagen) as per the manufacturer’s protocol. Briefly, the pellet was suspended in buffer RLT. The cells were sonicated and centrifuged, and the lysate was collected. One volume of 70% ethanol was mixed with the lysate, and the mixture was promptly transferred onto the RNeasy mini spin column. The column was then centrifuged for 15 s at 8,000*g*, and the flow-through was discarded. Subsequently, the column was washed with buffer RW1 and buffer RPE. Finally, RNA was eluted from the column with RNase-free water. The eluted RNA (1 μg) was treated with DNase I and subjected to cDNA synthesis using RevertAid H minus reverse transcriptase (Thermo Scientific) with random hexamer. cDNA prepared was used for RT-qPCR which was performed using TaqMan assay. ΔΔCT values of each strain were calculated using 16S rRNA as control.

### LexA cleavage assay

LexA cleavage assay was performed as per the published protocol (75, 76). The reaction was carried out in a 10-µL reaction mixture. 1 µM MsRecA was first incubated with shown amount of c-di-AMP for 5 min at 37 °C followed by the addition of 20 mM Tris–Cl, pH 7.0, 8 mM MgCl2, 1 mM DTT, 3 mM ATPϒS, and 5 µM SS DNA (63-mer oligonucleotide, HJ03). The reaction mixture was further incubated at 37 °C for 5 min followed by the addition of 3 µM LexA, and the reaction was carried out for 5 min at 37 °C. The cleavage was assessed by running the reaction mixtures on SDS–PAGE and Coomassie staining.

## Supplementary Material

pgae555_Supplementary_Data

## Data Availability

The data that support the findings presented in the manuscript are included in the manuscript and supplementary information.

## Preprints

This manuscript was posted on a preprint: https://doi.org/10.1101/2024.05.13.593841.
