# Supplementary material for: Cyclic di-AMP regulates genome stability and drug resistance in Mycobacterium through RecA-dependent and RecA-independent recombination
Source: PNAS Nexus. 2024 Dec 12;3(12):pgae555. doi: 10.1093/pnasnexus/pgae555 (PMC11653572; doi:10.1093/pnasnexus/pgae555)
Supplement: pgae555_Supplementary_Data [file pgae555_supplementary_data.docx]

**Cyclic di-AMP regulates genome stability and drug resistance in Mycobacterium through RecA-dependent and -independent recombination**

Sudhanshu Mudgal^1^, Nisha Goyal^1^, Kasi Manikandan^2^, Saginela Rahul^1^, Anusha Singhal^1^, Soumyadeep Nandi^3^, A K M Firoj Mahmud^4^, K. Muniyappa^2^, Krishna Murari Sinha^1*^

1. Amity Institute of Biotechnology, Amity University Haryana, Manesar, Gurgaon, Haryana 122413.
2. Department of Biochemistry, Indian Institute of Science, Bangalore 560012.
3. Umeå Plant Science Centre, Department of Plant Physiology, Umea University, 901 87 Umeå, Sweden.
4. CLINTEC, Karolinska Institutet, Alfred Nobels alle 8, 141 52 Huddinge, Stockholm, Sweden

*For correspondence: [kmsinha@ggn.amity.edu](mailto:kmsinha@ggn.amity.edu) OR [krishnamurarisinha2012@gmail.com](mailto:krishnamurarisinha2012@gmail.com)

**Table S1: SNPs detected in *∆disA M. smegmatis* cells:** Genome sequence of WT and *ΔdisA* *M. smegmatis* cells were compared and detected SNPs have been shown. ‘Position’ denotes the nucleotide position in reference *M. smegmatis* MC^2^ 155 sequence that has been mutated in *ΔdisA* cells denoted by ‘Observed Seq_*∆disA*’.

| **Position** | **Mutation type** | **Reference Seq_WT** | **Observed Seq_WT** | **Observed Seq_*∆disA*** | **Gene ID** | **Locus tag** | **Protein** | **Mutation in *∆disA*** |
| --- | --- | --- | --- | --- | --- | --- | --- | --- |
| 2223889 | ins | ACC | ACCC | ACC | cds-ABK71597.1 | MSMEG_2148 | HNH endonuclease  domain protein | ORF restored |
| 659940 | mnp | GA | GA | AG | cds-ABK72728.1 | MSMEG_0582 | Succinic semialdehyde  dehydrogenase | E306R |
| 665861 | snp | A | A | G | cds-ABK76014.1 | MSMEG_0589 | L-rhamnose isomerase | D315G |
| 779875 | snp | G | G | A | cds-ABK70539.1 | MSMEG_0692 | Molecular chaperone, putative | Silent mutation |
| 3105782 | complex | AT | AT | AGTG | cds-ABK72086.1 | MSMEG_3033 | 3-dehydroquinate synthase | termination codon  (truncated protein) |
| 3257230 | snp | T | T | C | cds-ABK73714.1 | MSMEG_3180 | transcriptional regulator,  MerR family protein | I46T |
| 3965123 | snp | T | T | G | cds-ABK72937.1 | MSMEG_3892 | Putative transcriptional  regulator | W62G |
| 5493870 | ins | ACG | ACG | ACCG | cds-ABK72913.1 | MSMEG_5414 | Septum formation initiator  subfamily protein, putative | termination codon  (truncated protein) |
| 6394806 | snp | C | C | A | cds-ABK70497.1 | MSMEG_6328 | tRNA adenosine deaminase | A73E |

**Table S2: Plasmids used in this study**:

| Name | Description | Source |
| --- | --- | --- |
| pRGM10 | Reporter construct, Plasmid carrying two inactive *lacZ* genes, [*lacZ(ISce-I)* and *∆NlacZ*], two ISce-I sites in opposite orientation, *attP* site, Km^R^ | [(Gupta *et al.*, 2011)](https://paperpile.com/c/yJeG2f/FS2cL) |
| pYS1 | A shuttle vector with a tm^S^ mycobacterial origin of replication, Che9c 60-61 protein, counter-selectable marker SacB, acetamide inducible promoter, Km^R^ | [(Shenkerman](https://paperpile.com/c/yJeG2f/zk7kX) [*et al.*, 2014)](https://paperpile.com/c/yJeG2f/zk7kX) |
| pYS2 | Plamsid carrying two *loxP* sites flanking the *gfp-hyg* cassette, Amp^R^(for *E. coli*), Hyg^R^ | [(Shenkerman](https://paperpile.com/c/yJeG2f/zk7kX) [*et al.*, 2014)](https://paperpile.com/c/yJeG2f/zk7kX) |
| pML2714 | Plasmid carrying tm^S^ mycobacterial origin of replication, express P1 Cre recombinase, Km^R^ | [(Shenkerman](https://paperpile.com/c/yJeG2f/zk7kX) [*et al.*, 2014)](https://paperpile.com/c/yJeG2f/zk7kX) |
| pdisAGFP | Plasmid carrying *M. tb disA* (*rv3586*) cloned in pVVGFP at HindIII and KpnI site, Km*^R^*, expresses DisA with C-terminal GFP tag | This work |
| pdisAGFP(2) | DisA mutated at D72A and G73A in pdisAGFP | This work |
| pRecA(1)gfp | Plasmid carrying *M. smegmatis recA* (*msmeg_2723*) cloned in pVVGFP at HindIII and KpnI site | [(Manikandan *et al.*, 2018)](https://paperpile.com/c/yJeG2f/njooR) |
| pEcRecA(1)gfp | Plasmid carrying *E. coli recA* cloned in pVVGFP at HindIII and KpnI site | [(Manikandan *et al.*, 2018)](https://paperpile.com/c/yJeG2f/njooR) |
| pVV17 | NdeI site in pVV16 mutated to SphI, Hyg^R^ | This work |
| pVVI-SceI | SphI-EcoRI digested *I-SceI* gene from pMSG375 cloned at similarly digested pVV17, Hyg^R^ | This work |
| pBSIntegrase | L5 integrase gene cloned at XhoI/EcoRI site of pBS (pBlueScript) plasmid | [(Manikandan *et al.*, 2018)](https://paperpile.com/c/yJeG2f/njooR) |
| pYS2MsRecA | Complementation construct for *M. smegmatis recA* (*msmeg_2723*), *msmeg_2723* along with hsp promoter amplified from pRecA(1)gfp and cloned in pYS2. | This work |
| pYS2EcRecA | Complementation construct for *E. coli recA* (*ecrecA*), *ecrecA* along with hsp promoter amplified from pEcRecA(1)gfp and cloned in pYS2. | This work |
| pYS2disA | Complementation construct for *M. tb disA*(*mtbdisA*), *mtbdisA* along with hsp promoter amplified from pdisAGFP and cloned in pYS2. | This work |
| pYS2disA(2) | Complementation construct for *M. tb disA* D72AG73A mutant in pYS2. | This work |
| pMsRecA1 | Plasmid carrying *M. smegmatis recA* cloned in pET28c between NdeI and XhoI sites, Km^R^ | (Manikandan *et al.*, 2018) |
| pEcRecA1 | Plasmid carrying *E. coli recA* cloned in pET28c between NdeI and XhoI sites, Km^R^ | (Manikandan *et al.*, 2018) |
| pLexA1 | Plasmid carrying *M. smegmatis lexA* cloned in pET28c between NdeI and HindII sites, Km^R^ | This work |
| pYS2radA | Plasmid used to create recombineering construct for deletion of *radA* (*msmeg_6079*) gene | This work |
| pYS2radAdisA | Plasmid used to create recombineering construct for deletion of both *radA* (*msmeg_6079*) and *disA* (*msmeg_6080*) genes together | This work |

Km^R^= kanamycin resistant, Amp^R^=Ampicillin resistant, Hyg^R^= Hygromycin resistant, tm^S^= Temperature sensitive, *gfp-hyg*= Green fluorescent protein and hygromycin resistant, SacB= Sucrose, DG= D72AG73A.

**Table S3: Bacterial strains used in this study:**

| Name | Description | Source |
| --- | --- | --- |
| *∆disA* | Derivative of *M. smegmatis* mc^2^ 155 carrying an unmarked deletion in *disA* | [(Manikandan *et al.*, 2018)](https://paperpile.com/c/yJeG2f/njooR) |
| *∆recA* | Derivative of *M. smegmatis* mc^2^ 155 carrying an unmarked deletion in *recA* | [(Manikandan *et al.*, 2018)](https://paperpile.com/c/yJeG2f/njooR) |
| *∆recA∆disA* | Derivative of *M. smegmatis* mc^2^ 155 carrying unmarked deletions in *disA* and *recA* | This work |
| mc^2^155:pRGM10 | mc^2^ 155 derivative carrying Km resistance plasmid vector pRGM10 integrated at the *attB* locus | This work |
| *∆disA*:pRGM10 | *∆disA* derivative carrying plasmid vector pRGM10 integrated at the *attB* locus | This work |
| *∆recA*:pRGM10 | ∆*recA* derivative carrying plasmid vector pRGM10 integrated at the *attB* locus | This work |
| *∆recA∆disA*:pRGM10 | *∆recA∆disA* derivative carrying plasmid vector pRGM10 integrated at the *attB* locus | This work |
| *∆recA*:*msrecA*:pRGM10 | Derivative ∆*recA* complemented with *M. smegmatis recA (msrecA*) at the intergenic region of *msmeg_5848* and *msmeg_5849* and plasmid pRGM10 integrated at *attB* locus | This work |
| *∆recA∆disA*:*msrecA*:pRGM10 | Derivative of *∆recA∆disA* complemented with *M. smegmatis recA (msrecA*) at the intergenic region of *msmeg_5848* and *msmeg_5849* and plasmid pRGM10 integrated at *attB* locus | This work |
| *∆recA*:*ecrecA*:pRGM10 | Derivative of ∆*recA* complemented with *E. coli recA* (*ecrecA)* at the intergenic region of *msmeg_5848* and *msmeg_5849* and plasmid pRGM10 integrated at *attB* locus | This work |
| *∆recA∆disA*:*ecrecA*:pRGM10 | Derivative of *∆recA∆disA* complemented with *E. coli recA* (*ecrecA)* at the intergenic region of *msmeg_5848* and *msmeg_5849* and plasmid pRGM10 integrated at *attB* locus | This work |
| *∆disA*:*mtbdisA*:pRGM10 | Derivative of *∆disA* complemented with *M. tb disA* (*mtbdisA)* at the intergenic region of *msmeg_5848* and *msmeg_5849* and plasmid pRGM10 integrated at *attB* locus | This work |
| *∆disA*:*mtbdisADG*:pRGM10 | Derivative of *∆disA* complemented with *M. tb disADG* (*mtbdisADG)* at the intergenic region of Msmeg_5848 and Msmeg_5849 and plasmid pRGM10 integrated at *attB* locus | This work |
| *∆radA*:pRGM10 | *∆radA* derivative carrying plasmid vector pRGM10 integrated at the *attB* locus | This work |
| *∆radA∆disA*:pRGM10 | *∆radA∆disA* derivative carrying plasmid vector pRGM10 integrated at the *attB* locus | This work |

**Table S4: List of primers used in this study:**

| Name | Primer Sequence (5’ to 3’) | Description |
| --- | --- | --- |
| C13F RecA  C14R RecA | TTTTTTCATATGGCGCAGCAGGCCCCAG  TTTCTCGAGTCAGAAGTCAACCGGGGCCG | To amplify *recA* (*msmeg_2723*) for cloning in pET28c |
| C15F LexA  C16R LexA | TTTTTCATATGATGAGCGACGACACCGGCGA  TTTAAGCTTTCAGATCTTGCGGATGACGGTG | To amplify *lexA* (*msmeg_2740*) for cloning in pET28c |
| C71F EcRecA  C72R EcRacA | TTTTTTCATATGGCTATCGACGAAAACAAACAG  TTTCTCGAGTTAAAAATCTTCGTTAGTTTCTGCTAC | To amplify *E. coli recA* (MG1655 Strain) for cloning in pET28c |
| C91F DisA  C92R DisA | TTTTAATTAACGTCGGCGACAACGAGACC  TTTATGCATCGAGCCAGCCCGAGTACGTC | Primers to amplify 3’ flanking homologous sequence for deleting *disA* gene |
| D49F MsRecA  D50R MsRecA | TTACTAGTACAGCAGGATGAGGTCGCCG  TTTTTTATTTAAATGTGCTCGGCGTCGATGAACG | Primers to amplify 5’ flanking homologous sequence for deleting *recA* gene |
| D51F MsRecA  D52R MsRecA | TTTTAATTAAGTCGAGCACGGCTTCATCCG  TTTATGCATCGACGCTGACCACGTCGTAG | Primers to amplify 3’ flanking homologous sequence for deleting *recA* gene |
| E16F  E17R | TTTTTTGCATGCAGAAAGGAGGCCATATGGG  TTTTGAATTCTTATTTCAGG AAAGTTTCGGAG | To amplify *I-SceI* from pMSG375 and cloning in pVV16 |
| E27F  E28R | GGAATCACTTCCATATGCCCAAGACAATTGCGGATC  GATCCGCAATTGTCTTGGGCATATGGAAGTGATTCC | To mutate NdeI and create a SphI site in pVV16. To mutate G388 to C in pVV16 and create pVV17a |
| E29F  E30R | CCGGAGGAATCACTTCCGCATGCCCAAGACAATTGC  GCAATTGTCTTGGGCATGCGGAAGTGATTCCTCCGG | To mutate AT385 to GC in pVV17a to create pVV17 |
| E37F  E36R | CACCGGATTCAGTCGTCACTC  CTCCAGTACAGCGCGGCTG | Anneals downstream of *kan* and N-terminal of lacZ of pRGM10. |
| E42F  E43R | GCTGATTCGAGGCGTTAACCG  TTCACCGCTTGCCAGCGGC | To confirm deletion after DSB repair. Binds across the break site in *lacZ(I-SceI)* |
| E55F  E56R | TGCCGATCGCGTCACACTAC  CAGACGCCACTGCTGCCAG | To confirm deletion after DSB repair. Binds across the break site in *lacZ(I-SceI)* |
| F19F  F21R | TTTTTTATTTAAATGGTGACCACAACGACGCGC  TTTTTTATTTAAATTTAAAAATCTTCGTTAGTTTCTGCTACG | To amplify *ecrecA* with hsp promoter from pEcrecA(1)gfp |
| F22R | TTTTTTATTTAAATTCAGAAGTCAACCGGGGCCGG | To amplify *msrecA* along with hsp promoter from pRecA(1)gfp |
| F23F  F24R | TTTTACTAGTCCCGTGCGGATCTTCCCC  TTTTTTATTTAAATCTAGGGTGCGTCGGTCAG | Primers to amplify 3' *msmeg_5848* |
| F33F | GAAAGCTGGCTACAGGAAGGC | Primer anneals downstream of 5’-region of pRGM10 |
| F48F  F49R | CTTCTCGCGCGTCGTCGCG  TGTCCTCGGCCCTCCGATC | To confirm the complementation, F48F anneals 721 bp upstream of the termination of msmeg_5848, F49R anneals at 107-125 nt of the hsp promoter |
| F58F  F59R | TTTTTAATTAACTAGGCCTGCAGCTTCTCGAAC  TTTTATGCATGATCAGGTGGGGCTGCGCG | Primers to amplify 3' *msmeg_5849* |
| F66F  F67R | TTTTTAATTAACGCGATGCCCATGAAGGCC  TTTTATGCATCGCTCGCCTGCCACGTAGAC | Primers to amplify 3’ flanking homologous sequence for deleting *radA* gene |
| F78F  F79R | TTTTACTAGTGTGGAGCTGACCAAGCCGATC  TTTTTTATTTAAATCCTCGAGCAGCAGCGTGGAC | Primers to amplify 5’ flanking homologous sequence for deleting *radA* gene |

F and R at the end of primer number indicate forward and reverse primer respectively**.**

**Methods:**

Whole genome sequencing (WGS) of the wild type (WT) and *ΔdisA M. smegmatis* cells was done at Macrogen, Seoul. Genomic DNA was extracted from both the strains and sequencing libraries were constructed by random fragmentation of the DNA followed by 5' and 3' adapter ligation. Adapter-ligated fragments were then PCR amplified and gel purified. Illumina sequencing platform was used to do paired-end sequencing of the samples with a read length of 151 bp. The BCL (base calls) binary was converted into FASTQ utilizing the illumina package bcl2fastq. We used the sequencing reads from the Illumina sequencing platform for the downstream bioinformatic analyses.

Single nucleotide polymorphisms (SNPs) for each sample, WT and *ΔdisA* were determined by aligning reads against the *Mycobacterium smegmatis* str. MC^2^155 as the reference genome and the variants were identified using the tool for fast bacterial variant calling from NGS reads Snippy (<https://github.com/tseemann/snippy>). Snippy v3.1 was used with the default setting to call the variants. Once the variants were identified, they were annotated by comparing the annotation file (GFF3 downloaded from <https://www.ncbi.nlm.nih.gov/Taxonomy/Browser/wwwtax.cgi?id=246196>) for the reference genome. The resulting annotated variants were compared against each other to identify the variations between the WT and *ΔdisA* genomes.

**Construction of single mutant *∆radA* and double mutant *∆radA∆disA M. smegmatis* cells:**

Deletion of genes in *M. smegmatis* was performed as previously described (Shenkerman *et al.*, 2014). This method generates unmarked deletion mutants in three consecutive steps. In the first step, *M. smegmatis* cells were electroporated with pYS1, a mycobacterial shuttle vector with a temperature-sensitive origin of replication and a selectable marker *sacB*. This plasmid contains the *Che9c 60-61* gene under the acetamidase promoter. The transformed cells were grown at 34°C against kanamycin in the presence of 0.2% acetamide to induce the expression of the Che9c 60-61 protein, and competent cells were prepared from them. These cells were then electroporated with the linear recombineering construct in the second step. This recombineering construct contains 5' and 3' flanking homologous sequences of the gene to be deleted. For deleting *radA* gene (*msmeg_6079*), the 5' flanking region of 545 bp was amplified with primers F78F and F79R, which include 336 bp from the coding sequence, and the 3' region, 715 bp long, was amplified with F66F and F67R primers, which included 231 bp of the open reading frame (ORF). This recombineering construct deleted 871 bp from the middle coding sequence of *radA (msmeg_6079)*. The construct contains a *gfp-hyg* cassette flanked by loxP sites. The electroporated cells were grown at 42°C in the presence of hygromycin and sucrose to cure the plasmid pYS1 and the GFP-positive cells were selected. The marked deletion of *radA* was also confirmed by genomic PCR amplification. To create an unmarked deletion of the gene, the cells were then transformed with pML2714 expressing P1 Cre recombinase, which removes the *gfp-hyg* cassette. The cells were further grown at 42°C to cure them of pML2714. Deletion of the *radA* gene was confirmed by genomic PCR and its sequencing.

The double mutant of *radA*(*msmeg_6079*) and *disA*(*msmeg_6080*) (*∆radA∆disA*) was performed similarly. The genes are present in an operon separated by only 36 bp. The 5' flanking region of the recombineering construct was amplified with F78F and F79R primers that were used to create *∆radA*. The 3' flanking region of 818 bp was amplified with C91F DisA and C92R DisA primers, which contain 411 bp of the ORF of *disA* and were used to delete *disA* (Manikandan *et al.*, 2018). This recombineering construct deleted a total of 1844 bp, 1083 bp and 725 bp from *radA* and *disA* ORF respectively.

**Construction of the double mutant *∆recA∆disA M. smegmatis* cells:**

The *recA*(*msmeg_2723*) gene was deleted in the previously constructed *∆disA M. smegmatis* cells (Manikandan *et al.*, 2018) to generate the double mutant *∆recA∆disA* following the procedure described above. Briefly, the recombineering construct contained a 5’ flanking sequence which was amplified with primers D49F MsRecA and D50R MsRecA. The product of 777 bp long which included 297 bp of the MsRecA ORF and the 3’ flanking sequence amplified with primers D51F MsRecA and D52R MsRecA is 710 bp long and contained 210 bp of the ORF. The construct deleted 543 bp from the ORF of *recA* and generated the double mutant *∆recA∆disA M. smegmatis* cells*.*

**Recombinant protein purification:**

The purification of the recombinant MsRecA (MSMEG_2723) has been described previously (Manikandan *et al.*, 2018). Briefly, the ORF encoding MsRecA was amplified through genomic PCR with forward and reverse primers C13F RecA and C14R RecA. The PCR product was digested with NdeI and XhoI and inserted into similarly digested pET28c, resulting in the construct pMsRecA1 that expresses MsRecA with an N-terminal His_6_ tag. The construct pMsRecA1 was transformed into Rosetta (DE3) *E. coli* cells. The cells were grown at 37℃ until A_600_≈ 0.5, chilled on ice for an hour, induced with 2% ethanol and 0.2 mM IPTG, and grown at 16℃ for 18 hours. The cells were harvested by centrifugation and the cell pellet was re-suspended in lysis buffer (buffer A) consisting of 50 mM Tris-HCl, pH 7.5, 0.25 M NaCl, 10% sucrose, 1 mg/mL lysozyme, and 0.1% Triton X-100 followed by incubation on ice for 45 minutes. The cell extract was sonicated, centrifuged, and the supernatant was collected.

Polymin-P was added to the lysate to a final concentration of 0.5%, followed by centrifugation. The pellet was resuspended in buffer A and centrifuged. The resulting pellet was extracted with buffer A containing 0.7 M NaCl and again centrifuged. The supernatant was subjected to ammonium sulfate precipitation, and the resulting pellet was dissolved in buffer B (50 mM Tris-HCl, pH 8.0, 0.25 M NaCl, 1 mM EDTA, 0.1% Triton X-100, and 10% glycerol). The solution was dialyzed against the same buffer for 18 hours.

The lysate was loaded onto a DEAE-Sephacel column pre-equilibrated with buffer B to remove the nucleic acids, and the protein was collected in the flow-through. This was dialyzed overnight in buffer B without EDTA and then applied to a Ni-NTA agarose column pre-equilibrated with the same buffer. The column was washed with buffer C (50 mM Tris-HCl, pH 8.0, 0.25 M NaCl, 0.05% Triton X-100, and 10% glycerol) and then with buffer C containing 50 mM, 300 mM, and 500 mM imidazole. The fractions were analyzed by SDS-PAGE, and MsRecA eluted in the 300 mM imidazole fractions. The protein was dialyzed against Buffer C containing 100 mM NaCl, aliquoted, and frozen at -80℃ for further use.

*E. coli* RecA (MG1655 Strain) was also purified using a similar procedure. The ORF encoding EcRecA was amplified with primers C71FEcRecA and C72REcRecA, cloned into pET28c to create pEcRecA1, and expressed with an N-terminal His_6_-tag. The protein was purified following the protocol used for MsRecA.

The *M. smegmatis lexA* gene (MSMEG_2740) was PCR amplified with primers C15F LexA and C16R LexA which have suitably placed NdeI and HindIII sites respectively. The amplified product was cloned into pET28c, resulting in pLexA1 which will express N-terminal His_6_ -LexA. The protein was expressed in Rosetta (DE3) *E. coli* cells by induction at 16°C as above. The cell pellet was suspended in buffer A and lysed by sonication. The lysate was applied to a Ni-NTA agarose column pre-equilibrated with buffer A, and washed with buffer B and buffer B containing 50 mM, 300 mM, and 500 mM imidazole. The protein eluted in buffer B containing 300 mM imidazole. The proteins were dialyzed against buffer C, applied to a DEAE-Sephacel column, and recovered in the flow-through. It was dialyzed in buffer B containing 100 mM NaCl, aliquoted, and frozen at -80℃.

**Methyl methane sulfonate (MMS) survivability assay:**

The sensitivity towards MMS was measured as previously described (Manikandan *et al.*, 2018). Briefly, the early log phase (A_600_ ≈0.5) cultures for both WT and *∆disA M. smegmatis cells* were centrifuged and resuspended in PBS with 0.05% Tween 80 followed by treatment with 0.5 % MMS at room temperature. 100 µl aliquots were then taken at specified times and 100 µl of ice-cold 10% sodium thiosulphate solution was added immediately to quench the reaction. Serial dilutions of the treated and untreated cells were then plated on 7H10 plates, and after 3 days, % survival was calculated compared with untreated control cells. The survival of the WT and *∆disA* cells was compared (Fig S2A).

**UV survival assay:**

UV irradiation assays were performed as described (Manikandan *et al.*, 2018). Briefly, *M. smegmatis* cells were grown to log-phase (A_600_ ≈0.5), and 10-fold serial dilutions were prepared and plated on 7H10 plates. The plates were exposed to the indicated UV doses in a CL-1000 ultraviolet crosslinker. Immediately after exposure, the plates were wrapped in foil to prevent repair by photolyase. Surviving colonies were counted after 3 days, and % survival was calculated compared with untreated control. The survival of the WT and *∆disA* cells was compared (Fig S2B).

**Sensitivity assay to ofloxacin, hydrogen peroxide, and rifampicin:**

The sensitivity of WT and *∆disA M. smegmatis* cells to ofloxacin, hydrogen peroxide, and rifampicin was determined as described before (Vandal *et al.*, 2009; Dupuy *et al.*, 2020). The cells were grown to early log phase (A_600_≈0.5) and incubated at 37°C for 2 and 4 h with 5 mM H_2_O_2_ (Merck), and 3 and 6 h with 5 µg/mL of ofloxacin. Serial dilutions of the treated cells were plated and CFU was calculated by normalizing with untreated cells. To measure sensitivity to rifampicin, serial dilutions were performed from 10^0^ to 10^−5^ in 7H9, and 100 µL of 10^-4^ dilution was plated on 7H10 or 7H10 supplemented with 10 µg mL^−1^ rifampicin. CFU was determined after a 4-d incubation at 37 °C and the percent survival was calculated for each strain (Fig S2C, D, and E).

**Western blotting**

Different strains of *M. smegmatis* were grown to early log phase (A_600_≈0.5) and incubated for an additional 3 hours at 37°C in the absence or presence of ofloxacin (10 µg/mL) to induce SOS response. The cells were harvested by centrifugation and resuspended in lysis buffer (50 mM Tris-HCl pH 7.5, 0.1 M NaCl, 10% glycerol, 10 mg/mL lysozyme, 0.1% Triton X-100, and 1 mM PMSF). The resuspended cells were incubated on ice for 30 min followed by sonication. The cell lysates (80 μg) of ofloxacin-treated and untreated cells were analyzed by Western blotting by probing with anti-RecA antibodies.

**Figures:**


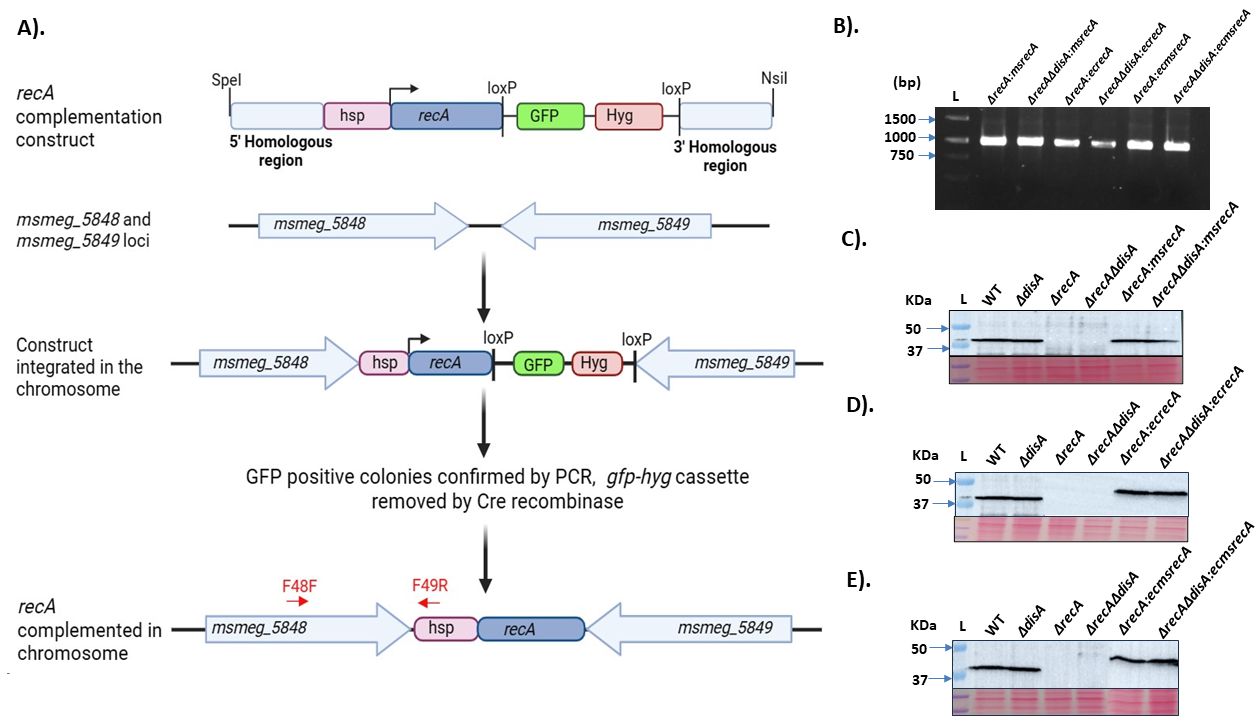


**Fig. S1 Complementation of mutants: A.** Construct for the complementation of *recA/disA* in *M. smegmatis*. Genes which are to be complemented have been placed under the heat shock promoter (hsp) for expression. The recombineering construct was integrated at the intergenic region of *msmeg_5848* and *msmeg_5849* in the genome. The *gfp-hyg* cassette was later removed by electroporating a plasmid expressing Cre recombinase. Integration of the construct was confirmed by doing PCR with primers F48F and F49R. The arrows show the orientation and position of the primers. **B.** Ethidium bromide-stained agarose gel showing the genomic PCR products of different strains as mentioned above the respective lanes. **C, D, and E.** Western blot showing the expression of RecA. The indicated strains of *M. smegmatis* were treated with ofloxacin (10µg/ml) to induce SOS response. Cell extract (soluble) was prepared and separated on 12% SDS-PAGE. The blot was probed with *anti-*RecA antibodies. The blot shown in C was probed with anti-MsRecA. D and E were probed with commercially available *E. coli* *anti*-RecA (Abcam 6379). The ponceau S-stained membrane shows equal loading of the extract.


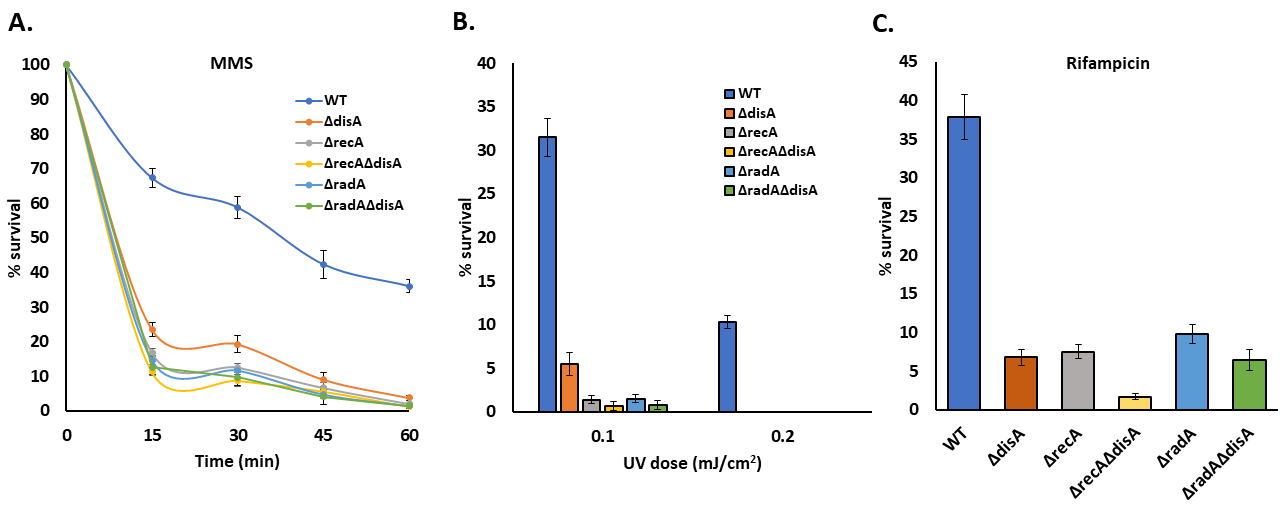


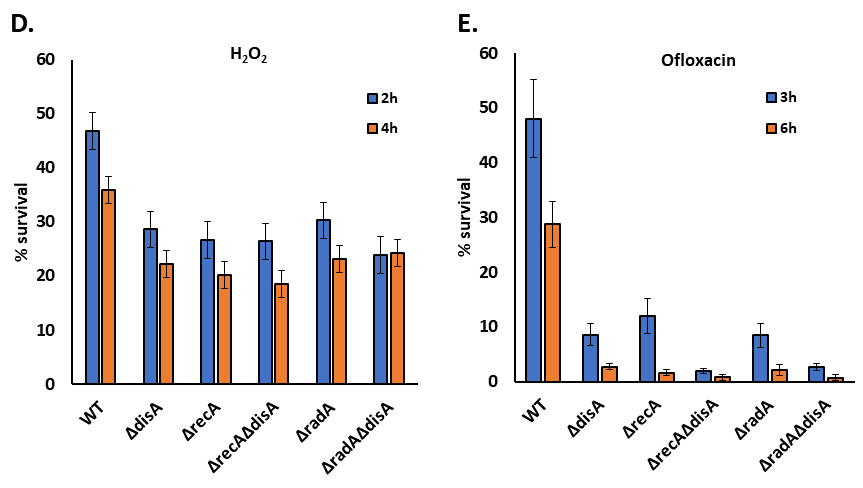


**Fig S2. Drug sensitivity assay. A.** The percent survival after treatment with MMS. The wild-type (WT), *∆disA*, *∆recA*, *∆recA∆disA*, *∆*radA, and *∆radA∆disA M. smegmatis* cells were treated with 0.5 % MMS for the indicated times and percent survival was calculated relative to the untreated cells. **B, C, D, and E.** Bar graphs representing the percent survival of WT, *∆disA*, *∆recA*, *∆recA∆disA*, *∆*radA, and *∆radA∆disA M. smegmatis* cells after treatment with the indicated doses of UV, 10µg/ml rifampicin, 5mM H_2_O_2_, and 5µg/ml ofloxacin respectively for the indicated time intervals. Each experiment was conducted with three biological replicates and each replicate was performed in triplicate.

**TAGGGATAACAGGGTAAT (N)_37_ ATTACCCTGTTATCCCTA**

**ATCCCTATTGTCCCATTA (N)_37_ TAATGGGACAATAGGGAT**

1. **Wild-Type**

**TACCATGGTAGGGATA Δ1/Δ1 TCCCTAAGCTTATC**

**ATGGTACCATCCCT ATAGGGATTCGAATAG**

**TACCATGGTAGGGATAAT /Δ2 CCCTAAGCTTATC**

**ATGGTACCATCCCTAT TAGGGATTCGAATAG**

**GATGGTGC Δ115/Δ4 CCCTAAGCTTATC**

**CTACCACG GGGATTCGAATAG**

1. ***∆disA***

**TACCATGGTAGGGATA Δ1/Δ1 TCCCTAAGCTTATC**

**ATGGTACCATCCCT ATAGGGATTCGAATAG**

**TACCATGGTAGGGAT Δ2/ TATCCCTAAGCTTATC**

**ATGGTACCATCCC TAATAGGGATTCGAATAG**

**TACCATGGTAGGGATAACA /Δ6 CTAAGCTTATC ATGGTACCATCCCTATTGT GATTCGAATAG**

**CATTATCC Δ57/Δ5 CTAAGCTTATC**

**GTAATAGG GATTCGAATAG**

**TATCGTGCGGTGGT Δ297/Δ219 AACTGCCTGAAC**

**ATAGCACGCCACCA TTGACGGACTTG**

1. ***∆recA∆disA***

**CATGGTAGGGATAACAGGG TATCCCTAAGCTT**

**GTACCATCCCTAT AATAGGGATTCGAA**

**Fig. S3. Molecular outcome of DSB repair by NHEJ:** The sequence at top are the two 18-mer I-SceI target sequences placed 37 nt apart in opposite orientation in *lacZ*(*I-SceI*). The sequence shown in red are of the DSB generated with 3’ non-complementary overhangs after cleavage by I-SceI. ∆n/∆n represents the number of nucleotides deleted from the 3’ overhangs during repair, inserted nucleotides have been shown in green. **A** and **B.** show the repair outcomes in wild-type and *∆disA* cells. **C.** shows the insertion of nucleotides at the break site, there is no deletion of nucleotides during repair.


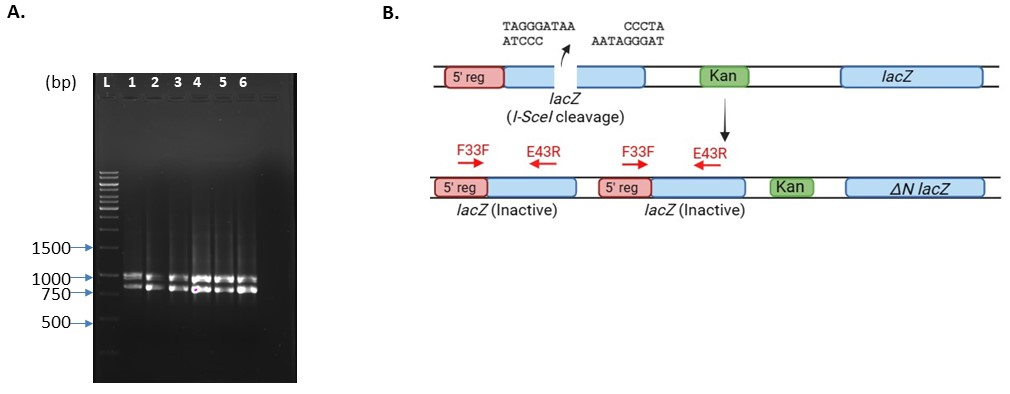


**Fig. S4 Duplication of *lacZ(I-SceI)* gene: A.** PCR product of six different white colonies obtained after streaking. Genomic PCR amplification of 6 white colonies was done with primers F33F and E43R. PCR product was run on agarose gel and visualized by ethidium bromide staining as shown. **B.** The upper panel shows the I-SceI cleaved reporter construct pRGM10 whereas the lower figure shows its repair outcome. Duplication of *lacZ(I-SceI)* gene giving rise to 2 inactive *lacZ* during repair has been shown.


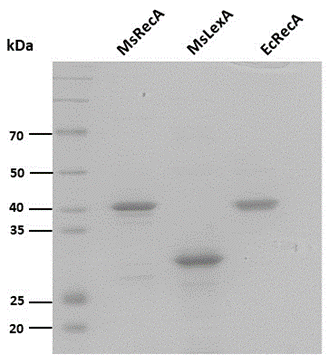


**Fig S5.** A Coomassie Blue-stained SDS-PAGE showing 5µg of the indicated purified recombinant proteins. The left-most lane shows the size and position of the molecular weight markers.


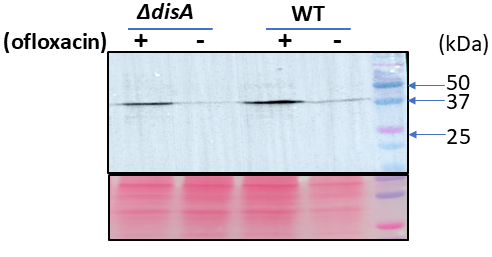


**Fig S6.** Western blot to show the expression of RecA in WT and *∆disA M. smegmatis* cells. The cells were treated with 10 µg/mL of ofloxacin to induce the SOS response. Soluble cell extracts were prepared and used for the Western blot analysis. The '+' and '–' lanes represent ofloxacin-treated and untreated cell extracts, respectively. The rightmost lane shows the molecular weight markers. The Ponceau S-stained membrane at the bottom shows equal loading of the extracts.

**References:**

Dupuy, P., Howlader, M., and Glickman, M.S. (2020) A multilayered repair system protects the mycobacterial chromosome from endogenous and antibiotic-induced oxidative damage. *Proc Natl Acad Sci* **117**: 19517–19527 https://pnas.org/doi/full/10.1073/pnas.2006792117.

Manikandan, K., Prasad, D., Srivastava, A., Singh, N., Dabeer, S., Krishnan, A., *et al.* (2018) The second messenger cyclic di‐AMP negatively regulates the expression of Mycobacterium smegmatis recA and attenuates DNA strand exchange through binding to the C‐terminal motif of mycobacterial RecA proteins. *Mol Microbiol* **109**: 600–614 https://onlinelibrary.wiley.com/doi/10.1111/mmi.13991.

Shenkerman, Y., Elharar, Y., Vishkautzan, M., and Gur, E. (2014) Efficient and simple generation of unmarked gene deletions in Mycobacterium smegmatis. *Gene* **533**: 374–378 https://linkinghub.elsevier.com/retrieve/pii/S0378111913013024.

Vandal, O.H., Roberts, J.A., Odaira, T., Schnappinger, D., Nathan, C.F., and Ehrt, S. (2009) Acid-Susceptible Mutants of Mycobacterium tuberculosis Share Hypersusceptibility to Cell Wall and Oxidative Stress and to the Host Environment. *J Bacteriol* **191**: 625–631 https://journals.asm.org/doi/10.1128/JB.00932-08.
